# A scoping review of de-implementation frameworks and models

**DOI:** 10.1186/s13012-021-01173-5

**Published:** 2021-11-24

**Authors:** Callie Walsh-Bailey, Edward Tsai, Rachel G. Tabak, Alexandra B. Morshed, Wynne E. Norton, Virginia R. McKay, Ross C. Brownson, Sheyna Gifford

**Affiliations:** 1 Prevention Research Center, Brown School, Washington University in St. Louis, One Brookings Drive, Campus Box 1196, St. Louis, MO 63130 USA; 2 Department of Surgery, Division of Public Health Sciences, Washington University School of Medicine, 660 S. Euclid Ave, St. Louis, MO 63110 USA; 3 Department of Behavioral, Social, and Health Education Sciences, Rollins School of Public Health, Emory University, 1518 Clifton Rd NE, Atlanta, GA 30322 USA; 4 Division of Cancer Control and Population Sciences, National Cancer Institute, 9609 Medical Center Drive, Bethesda, MD 20850 USA; 5 Department of Surgery (Division of Public Health Sciences) and Alvin J. Siteman Cancer Center, Washington University School of Medicine, 4921 Parkview Place, Saint Louis, MO 63110 USA; 6 Department of Physical Medicine and Rehabilitation, Washington University in St. Louis, 4444 Forest Park Ave, Campus Box 8518, St. Louis, MO 63108 USA

**Keywords:** De-implementation, De-adoption, Disinvestment, Obsolescence, Low-value, Health, Model, Framework, Scoping review

## Abstract

**Background:**

Reduction or elimination of inappropriate, ineffective, or potentially harmful healthcare services and public health programs can help to ensure limited resources are used effectively. Frameworks and models (FM) are valuable tools in conceptualizing and guiding the study of de-implementation. This scoping review sought to identify and characterize FM that can be used to study de-implementation as a phenomenon and identify gaps in the literature to inform future model development and application for research.

**Methods:**

We searched nine databases and eleven journals from a broad array of disciplines (e.g., healthcare, public health, public policy) for de-implementation studies published between 1990 and June 2020. Two raters independently screened titles and abstracts, and then a pair of raters screened all full text records. We extracted information related to setting, discipline, study design, methodology, and FM characteristics from included studies.

**Results:**

The final search yielded 1860 records, from which we screened 126 full text records. We extracted data from 27 articles containing 27 unique FM. Most FM (*n* = 21) were applicable to two or more levels of the Socio-Ecological Framework, and most commonly assessed constructs were at the organization level (*n* = 18). Most FM (*n* = 18) depicted a linear relationship between constructs, few depicted a more complex structure, such as a nested or cyclical relationship. Thirteen studies applied FM in empirical investigations of de-implementation, while 14 articles were commentary or review papers that included FM.

**Conclusion:**

De-implementation is a process studied in a broad array of disciplines, yet implementation science has thus far been limited in the integration of learnings from other fields. This review offers an overview of visual representations of FM that implementation researchers and practitioners can use to inform their work. Additional work is needed to test and refine existing FM and to determine the extent to which FM developed in one setting or for a particular topic can be applied to other contexts. Given the extensive availability of FM in implementation science, we suggest researchers build from existing FM rather than recreating novel FM.

**Registration:**

Not registered

**Supplementary Information:**

The online version contains supplementary material available at 10.1186/s13012-021-01173-5.

Contributions to the literature

- This study provides an overview of previously published frameworks and models used to study de-implementation of interventions and policies from a wide array of disciplines, including healthcare, public health, and public policy.
- The frameworks and models identified can be applied to future studies of de-implementation of ineffective, contradicted, mixed, or untested health care practices or public health policy.
- This study highlights multiple gaps in de-implementation research and suggests actions to advance future work in the field.

## Background

Low-value and inappropriate medical care is recognized as a costly “wicked problem” in need of remedy [1]. In 2012, the Institute of Medicine (now the National Academy of Medicine) estimated that wasteful spending contributed to one-third of healthcare costs in the USA, and over $200 billion was attributed to unnecessary care [2, 3]. A 2018 report from the Organization for Economic Co-operation and Development (OECD) indicated that 20% of healthcare spending in the European Union was wasteful [4]. Another report stated that correcting for inappropriate or harmful practices accounts for over 10% of hospital spending in OECD member nations globally [5], indicating valuable resources are not being allocated efficiently.

Overuse or inappropriate use of screening, diagnostic services, and treatments contribute to patient harms including overdiagnosis and contraindicated treatments, unnecessary treatment and financial burden on patients, increased risk of adverse outcomes, and worsened care quality [6, 7]. A 2019 meta-analysis of studies published in three prominent medical journals found 396 reversals of medical practices [8], suggesting healthcare is rife with opportunities to eliminate ineffective or harmful practices. In public health, a classic example of an ineffective intervention is the D.A.R.E. program, which, despite evidence suggesting the program is ineffective and possibly counterproductive [9–11], is still offered in all US states and in more than 50 countries globally [12]. Another example is abstinence-only education models for sexual health, which despite evidence for the ineffectiveness and potential harms of these approaches, are still prevalent [13]. In Uganda, for example, evidence suggests abstinence only education was not effective in reducing the risk of sexually transmitted diseases, and numerous studies suggest detrimental impacts of such programs on sexual health [14–16]. Given there is far less funding in public health than in medicine [17], particularly in resource-limited settings, waste resulting from the continuation of ineffective programs is even more costly.

Low-value care also has implications for health equity. Helfrich and colleagues highlight several examples of racial and ethnic disparities in both overuse and underuse [18]. For instance, Black and Hispanic patients receive a higher rate of low value care than white patients for several services, such as inappropriate feeding tube use among dementia patients [19]. Furthermore, patients of color with low socioeconomic status subsidize low-value care among more affluent white patients [20]. When overprescribing or overuse occurs, this leaves fewer resources that can go to patients in need [21]. McKay and colleagues suggest it is unethical to leave harmful, ineffective, or unnecessary interventions in place when removal and/or replacement is warranted, and at the same time caution researchers to carefully consider the contextual factors surrounding these interventions and potential remedies as to not further disempower marginalized stakeholders [22].

These findings and others have helped bolster the importance of de-implementation research to develop approaches to promote the reduction of unnecessary interventions. De-implementation is defined as discontinuing or abandoning practices that are not proven to be effective, are less effective or less cost-effective than an alternative practice, or are potentially harmful [22, 23]. In public policy, “termination” is analogous to de-implementation, and refers to the “deliberate conclusion or cessation of specific government functions, programs, policies, or organizations,” [24] and unlike in implementation science, is agnostic about the weight and direction of evidence of effectiveness. This, along with other concepts from public policy, such as “disinvestment”, are particularly relevant to the study of de-implementation because they characterize in depth the context surrounding termination or disinvestment (e.g., landscape of risk to policymakers, constituent and political pressures, choices of strategy), policy characteristics that facilitate or hinder termination, and organizational termination processes [25–27]. Decades of research scattered across healthcare, public health, public policy, and business have been devoted to investigating efficient, effective means for removing unnecessary programs and phasing out policies that are no longer useful or relevant. However, the formal study of best practices in systematic cessation (i.e., de-implementation) remains a relatively new area of inquiry within the field of implementation science and has room for significant development [28].

Despite greater attention to the importance of reduction of overuse, there is a lack of guiding frameworks for this work to inform research, data collection, and analysis, and to generate scientific consensus [6]. Harris and colleagues conducted a literature review of disinvestment in health services; they did not find models for systematic organizational approaches to evidence-based decision making regarding disinvestment [29]. Although implementation science has an abundance of frameworks and models to guide the study of implementation, it is unclear if these are suitable guides for de-implementation research [30] given differences in processes involved and necessary behavior change strategies [28, 31]. A recent review of de-implementation studies published in 2013-2018 found ten theories, models, and frameworks to guide or explain de-implementation of low-value care [32]. Although this review provides helpful information for the field, it was limited in scope to five years of publications and was aimed mainly towards medical care. Decades of research on policy termination illustrate the potential to learn about de-implementation or discontinuance of practices from an array of disciplines, presenting an important opportunity to expand upon this work.

Given the importance of effective de-implementation to health outcomes and paucity of information about frameworks and models that can be applied to de-implementation, we conducted a scoping review to achieve the following aims: (1) To identify published frameworks and models that can be used to study de-implementation as a phenomenon; (2) To map each framework and model’s stated scope according to discipline, purpose, comprehensiveness, and measurement; and (3) To identify gaps in the literature to inform future model development and application for research. Our current review expands on previous efforts by including thirty years of publications from a broad array of relevant fields (e.g., healthcare, public policy, business), databases, and peer-reviewed journals. In the current review, frameworks are defined as “a set of variables and the relationships among them that are presumed to account for a set of phenomena.” [33] Models are conceptualized as more specific than frameworks and are “used to make specific assumptions about a limited set of parameters and variables” [33]. This review is explicitly focused on visual representations of frameworks and models (FM from here on) that may be used as a guide for researchers and practitioners in designing, executing, and evaluating de-implementation efforts.

## Methods

### Search strategy

We conducted a systematic search and scoping review following the Preferred Reporting Items for Systematic Reviews and Meta-Analyses (PRISMA-ScR) guidelines for scoping reviews [34]. The authors reviewed canonical articles and consulted with a research librarian to develop search terms. A research librarian specializing in literature review assisted in searching published literature for the concepts of “de-implementation,” “models” and “frameworks,” and “use patterns.” Due to the broad nature of search terms used to capture these concepts, the search was optimized over multiple iterations. With each iteration, increased precision was achieved by utilizing controlled vocabulary terms, proximity searching, and keywords. In order to capture the maximum number of potentially relevant FM, nine databases were included in the search: Ovid Medline, Embase, Scopus, Cumulative Index of Nursing and Allied Health Literature (CINAHL), Econlit, Global Health, APA PsycInfo, SocINDEX, and Cochrane. The search was limited to studies published in English from 1990 to June 2020 and did not include animal studies. The primary search resulted in 6,253 records. From this, incremental searches with journal limiters on the primary search and hand searching of eleven relevant journals for study protocols resulted in an additional 141 records (Supplemental File 1). Records were entered into EndNote reference management software for deduplication.

### Inclusion and exclusion criteria

At the title and abstract screening phase, we applied broad inclusion criteria such that any article that used a de-implementation related term from our search strategy and mentioned a framework or model was included for full text review. Studies were excluded if they were not published in English, published prior to 1990, or did not include relevant terms in the title or abstract. We applied additional exclusion criteria at the full text screening phase, such that records were excluded if the full text record was irretrievable, the article did not include a visual representation of FM, the FM depicted were not relevant to de-implementation (e.g., indicated discontinuance of therapy upon patient recovery), or the search terms were used in a different context (e.g., “model” referred to a statistical model). We did not explicitly limit to strategies to support de-implementation (analogous to implementation strategies); we also included de-implementation processes, efforts, and projects broadly.

### Screening procedures

Two raters (CWB and SG) conducted a pilot title and abstract screening with approximately five percent of records. A selected subset of records were brought to the full team for discussion and review to inform screening decisions. Once consensus was achieved, the two raters conducted independent title and abstract screenings. For cases where a clear decision could not be made, the raters deliberated with the full team to reach a decision.

Once the final set of records was identified for full text screening, the first author developed screening procedures based on team input. Two raters (CWB and ET) piloted the screening procedures with a randomly selected subset of 15 full text records. The study team refined the procedures and the two raters piloted a second set of randomly selected 15 full text records. Upon finalizing the screening procedures and reaching consensus, the two raters conducted dual independent screening of the remaining full text records. The raters met weekly for consensus. When consensus could not be reached, the raters consulted the full team for a final decision. By using a consensus coding approach, raters reached 100% agreement, thus an interrater reliability statistic was not calculated. The raters used a hierarchical exclusion approach such that the first applicable exclusion reason was applied (see Supplemental File 1 for exclusion codes). The team then conducted a subsequent hand-search of relevant references from articles included at the full text screening phase. This hand-searching approach primarily served to attempt to locate articles providing a visual representation of FM referenced in full text articles included in our review. We did not identify any additional articles that met the criteria to consider for full text review.

### Data extraction

The first author developed a data extraction procedures manual incorporating team input. The constructs selected for extraction and coding were informed by previous reviews and conceptual articles in FM and de-implementation published by team members [22, 30, 35]. Two raters (CWB and ET) extracted descriptive information such as country and setting of study, topic area, and the subject of de-implementation (e.g., medical intervention, organizational practice, public policy). To capture how this topic is described in the literature, the raters extracted terms relevant to de-implementation. The raters then classified the de-implementation action targets of the FM based on the four categories presented by Norton and Chambers [35]: *reduce, replace, restrict, remove*. *Reduce* refers to the decrease in use (frequency, intensity) of an intervention. *Replace* indicates eliminating an inappropriate intervention and putting a new evidence-based program targeting the same or similar outcomes in its place. *Restrict* narrows by whom, where, and/or for whom an intervention is used. *Remove* occurs when an inappropriate intervention is eliminated from practice or a policy is terminated (without replacement).

We classified the evidence for de-implementation presented by included studies according to Norton and Chambers [35]. We coded a study as presenting supporting evidence that an intervention was *ineffective* if the authors cited studies demonstrating that the intervention does not improve patient outcomes or causes harm to patients. *Contradicted* evidence indicated that more recent or higher quality studies suggest that an intervention previously suggested to be beneficial may in fact be ineffective or harmful. *Mixed* evidence indicated that the quality and quantity of evidence available to support the effectiveness, or lack thereof, and/or harmfulness of an intervention is roughly equal. *Untested* suggested that the intervention has not yet been tested for effectiveness. If a study did not cite specific evidence for the de-implementation of a practice or policy, we coded this as not reported (NR).

To determine how de-implementation efforts are currently studied, we extracted information on study design, methodology, measurement, and outcomes of interest. FM characterization was informed by previous reviews on implementation models and frameworks [30, 32] and included model name, type of framework or model (i.e., determinants model, evaluation framework, process model, theoretical framework; see Supplemental File 2 for definitions), and the nature of relationships between constructs (i.e., linear; cyclical/feedback; nested). As in the previous review by Tabak and colleagues [30], we classified FM according to the socio-ecological framework (SEF) [36]. Recognizing that personal-level processes related to de-implementation can be within an individual (e.g., cognitive processes such as unlearning [37]) or involve interactions between individuals (e.g., shared decision-making between a patient and provider), we expanded our classification system to distinguish the individual level into “interpersonal” and “intrapersonal.” The coders’ approach to classifying the FM per the levels of the SEF relied on the authors’ descriptions of how the FM was applied in their study (for empirical articles) or the descriptions of FM development and application (for conceptual articles). Coders did not make inferences beyond what was available in the text or draw upon other background knowledge or text to assign levels of the SEF.

Two raters (CWB and ET) piloted the extraction procedures on two articles and met with the study team to revise. The final extraction procedures and extraction variables were entered into a Google Sheets extraction database. Two raters (CWB and ET) conducted dual non-independent data extraction such that a primary rater would highlight relevant text in the article and enter data into the database and the second rater would review for accuracy and completeness. The raters met weekly to generate consensus. As before, when consensus could not be achieved, the full team was consulted. Upon completing data extraction, the first author generated descriptive statistics (e.g., frequencies) and qualitative descriptions of the data, reviewed by the co-authors for accuracy. The protocol for this review is not registered with a review tracking database; however, a detailed protocol is available from the authors upon request.

### Quality assessment

To assess the quality of the included empirical articles (*n* = 13), the first author applied the Mixed Methods Appraisal Tool (MMAT) [38], which allows for the assessment of a wide array of study designs. The MMAT includes two initial screening questions applicable to all study types to assess minimum quality for inclusion. Included studies are rated “yes”, “no”, or “can’t tell” on five questions specific to the type of study design (i.e., qualitative studies are rated on different criteria than quantitative designs). The MMAT authors do not recommend excluding studies based on poorer quality ratings or creating quality thresholds based quantitative ratings [38], thus we have not taken such an approach in our quality assessment. Due to the nature of the quality rating criteria, it was not possible to assess the quality of review studies and conceptual articles not involving data collection and analysis.

## Results

### Yield

**Fig. 1:**
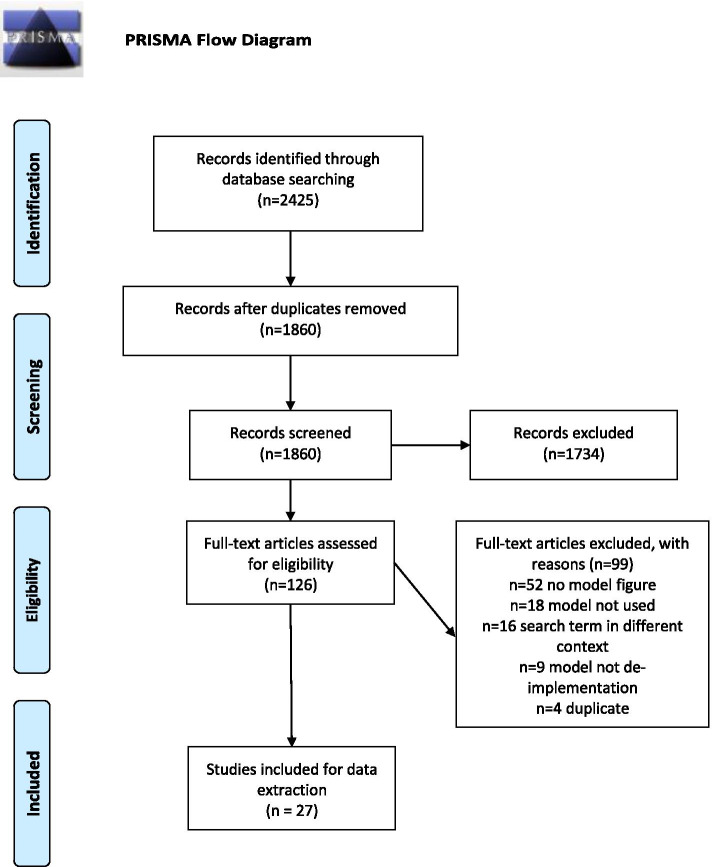
PRISMA flow diagram

The final search yielded a total of 1860 unique records after deduplication. During the title and abstract screening phase, 1734 records were excluded, leaving 126 records for full text screening. During full text screening, 99 records were excluded, most frequently due to no visual depiction of a model or framework referenced in the text (*n* = 52). A total of 27 full text records were included for final data extraction (see PRIMSA-ScR Figure 1).

### Study characteristics

The 27 published articles extracted in the final search and included in this review were nearly-even split between empirical (*n* = 13; see Table 1) and non-empirical studies (*n* = 14; see Table 2). Mixed methods study designs (*n* = 11) were most common among empirical studies. Only two quantitative studies were included: one of which utilized a cross-sectional longitudinal design [49], the other of which was a randomized controlled trial [50]. No qualitative empirical studies were included in this review. Of the non-empirical studies, 11 were commentaries or conceptual pieces; two were review studies. Twenty of the 27 included studies reported where the study took place. All were based in OECD countries, including: the USA (*n* = 9), Australia (*n* = 4), and multiple countries within the OECD (*n* = 3). The majority of studies that met the final criteria for inclusion were from the healthcare or public health sector (*n* = 22). Three were from organizations across multiple sectors and two were from public policy broadly. The median number of MMAT criteria met by included empirical studies was 4 (out of 5). More detailed reporting of study quality is available in Supplemental File 3.

**Table 1:** Study description of empirical studies (*N* = 13)

Author, year	Setting	Sample characteristics	Topic/content area	De-implementation intervention	Primary action	Secondary action	Evidence for de-imp.	Cost	Stakeholder	Method	Study design	Measures used	Primary outcomes
Cuttler et al., 2005 [39]	Clinical (academic, specialty)	Physicians (*n* = 222)	Pediatric specialty medicine	Physician decision to terminate growth hormone therapy in pediatric patients	Reduce, remove	NA	Mixed	Y	Y	Mixed methods	Cross-sectional	Interview; survey	Physician recommendation for case Scenarios
Goodwin, 2013 [40]	Healthcare (broad)	Healthcare manager, staff, and clinicians (*n* = 13)	Reducing low-value care and costs in healthcare	Program budgeting and Marginal analysis	Reduce	NA	NR	Y	Y	Mixed methods	Case study	Interviews; archival data	Satisfaction and compliance with pbma process; cost
Grimshaw et al., 2020 [41]	Healthcare (broad)	Hospitals (*n* = 137)	Preoperative tests; imaging for lower back pain	Identify low-value care; identify local priorities; identify barriers and potential interventions; evaluate choosing wisely implementation; spread effective choosing wisely programs	Reduce	NA	Ineffective	Y	Y	Mixed methods	Case study	Hospital administrative data	% reduction in low-value care
Gupta et al., 2019 [42]	Hospital	Patient records (*n* = 4,781)	Neutropenic diet for immunocompromised patients	Multi-step implementation strategy bundle targeting clinician and system-level change (e.g., training, EHR updates)	Remove	NA	Ineffective	Y	Y	Mixed methods	Case study	Content analysis of neutropenic diet prescribing; EHR review	Absolute reduction in prescribing
Harris et al., 2017a [29]	Health service network	Healthcare experts, steering committee members, workshop attendees (*n* = 28)	Disinvestment of clinically or cost ineffective health services (broadly)	Development of deimplementation framework	Remove	NA	NR	Y	Y	Mixed methods	Case study	Literature review; interview; survey; workshop	Development of deimplementation framework
Harris et al., 2017b [43]	Health service network	Healthcare experts (*n* = 15), healthcare staff (*n* = 65), senior administrators (*n* = 18)	Disinvestment of clinically or cost ineffective health services (broadly)	Development of deimplementation framework	Remove	NA	NR	Y	Y	Mixed methods	Case study	Literature review; interview; survey; workshop	Describe methods for disinvestment
Harris et al., 2017c [44]	Health service network	Healthcare experts (*n* = 15), healthcare staff (*n* = 65), senior administrators (*n* = 18)	Disinvestment of clinically or cost ineffective health services (broadly)	Development of deimplementation framework	Remove	NA	NR	Y	Y	Mixed methods	Case study	Literature review; interview; survey; workshop	Development of deimplementation framework
Harris et al., 2018 [45]	Health service network	NA	Disinvestment of clinically or cost ineffective health services (broadly)	Development of deimplementation framework	Remove	NA	NR	Y	Y	Mixed methods	Case study	Literature review; interview; survey; workshop	Development of deimplementation framework
McKay et al., 2017 [46]	Non-profit community based organization	Organization staff (*n* = 5); clients (*n* = 396)	HIV prevention	Counseling intervention to identify and reduce risk behaviors. De-adoption involved transition of resource to replacement intervention.	Remove	Replace	Ineffective	Y	N	Mixed methods	Archival secondary data analysis, interview	Data abstraction of agency archival and client records, interviews (with program staff)	Intervention deadoption process and consequences
Padek et al., 2018 [47]	State health departments	Program staff and leaders (n not available in protocol)	Cancer prevention and control programs in public health departments	NR	Reduce	NA	Ineffective	Y	Y	Mixed methods	Study protocol (quantitative crosssectional, qualitative case study, abm simulation)	Survey, case studies (interview), abm	Mis-implementation of cancer prevention and control programs
Skolarus et al., 2018 [48]	Clinical (VA)	Patients and physicians (n not available in protocol)	Prostate cancer (androgen deprivation therapy)	Organization policy and behavior change (assess preferences and barriers; discrete choice experiment; formulary restriction; strategy targeting patient/provider decision making)	Reduce	Restrict	Ineffective, contradicted	N	Y	Mixed methods	Study protocol (crosssectional)	Interviews, surveys	Acceptability, feasibility, scalability
Tangpong et al., 2015 [49]	Organization/firm (multiple industries)	Organizations (*n* = 96)	Business/management	Organizational behavior, layoffs, divestments, geographic exits	Reduce	Remove, restrict	NR	Y	N	Quantitative	Longitudinal	Survey	Likelihood of turnaround success, changes in form operating conditions, internal firm performance, external capital market support
Voorn et al., 2018 [50]	Hospital	Hospitals (*n* = 21)	Patient blood management in transfusion medicine (surgery)	Information provision, goal specification, clinician feedback, benchmark with comparison to best practice hospitals (behavior change)	Reduce	NA	Ineffective	Y	Y	Quantitative	Cluster randomized control trial	Survey	Use of low- value care (esa + blood salvage)

*Note*: *ABM* agent-based modeling, *N* no, *NA* not applicable, *NR* not reported, *Y* yes. For the purposes of this review, “empirical” is defined as collection and analysis of primary data

**Table 2:** Description of non-empirical and review studies (*N* = 14)

Author, year	Setting	Topic/content area	De-implementation intervention	Primary action	Secondary action	Evidence for de-imp.	Cost	Stakeholder	Method	Study design
Amankwah-Amoah, 2017 [51]	Industries (broad)	Technological lifecycle	Describe the lifecycle of a product, ultimately leading to discontinued use of a technology	Remove	Replace	NR	Y	N	Non-empirical	Commentary
Bain et al., 2008 [52]	Clinical	Medication discontinuation	Recognize indication for discontinuing medication; identify medication for discontinuation; discontinue; monitor patient	Remove	NA	Ineffective	Y	Y	Non-empirical	Case example; commentary
Bauer, 2014 [53]	Legislative bodies (broad)	Environmental (air pollution, water protection, wildlife protection); social (child benefits, pension, unemployment) policy change	Public policy dismantling (including strategies)	Remove	NA	NR	Y	N	Non-empirical	Literature summary; commentary
Davidson et al., 2017 [54]	Clinical	Behavior change	Broad cycle of identifying low-value practice, plan and execute de-implementation, evaluate consequences, plan for new practice implementation	Remove	Replace	NR	Y	Y	Non-empirical	Commentary
Helfrich et al., 2018 [37]	Clinical	De-implementation of low-value or ineffective clinical practices (broadly)	Unlearning an active process and substitution of an alternative practice	Reduce; replace	NA	Ineffective	N	Y	Non-empirical	Commentary
Herald et al., 2009 [55]	Systems (broad, primary example military)	Technological lifecycle	Discuss 6 components of obsolescence management: technology road mapping, system costing, system obsolescence life cycle forecasting, technology trade study analysis and product selection, technology/product surveillance and health assessment, technology transition	Replace	NA	NR	Y	N	Non-empirical	Commentary
Hyun-Ju et al., 2016 [56]	Healthcare system (broad)	Healthcare technology reassessment	Strategies to reassess and manage obsolete medical technologies	Reduce; replace	Remove	Mixed	Y	Y	Review	Systematic review
Kirkpatrick et al., 1999 [26]	Public policy	Public policy termination	Consider political environment, policy characteristics, and system constraints	Reduce, remove	NA	NR	Y	Y	Non-empirical	Conceptual
Niven et al., 2015 [57]	Clinical	Low-value clinical practices	Selected and tailored deadoption intervention	Remove	Reduce	Ineffective, contradicted	Y	Y	Review	Scoping review
Norton et al., 2019 [58]	Clinical	Cancer care delivery	De-implementation strategies (at patient, provider, setting, societal levels)	Reduce, remove replace, restrict	NA	Ineffective, contradicted, mixed, untested	N	N	Non-empirical	Commentary
Prasad, 2014 [23]	Healthcare system (broad)	Contradicted, untested, novel healthcare practices	Factor prioritization for testing unproven practices	Remove	NA	Untested	Y	N	Non-empirical	Commentary
Scott et al., 2013 [59]	Clinical	Polypharmacy in older populations	Behavior change (begin using discontinuation guide) assess for deprescribing	Remove	NA	Ineffective, contradicted	Y	Y	Non-empirical	Commentary
Soril et al., 2020 [60]	Healthcare system (broad)	Healthcare technology	Policy process (selection, decision, execution, reassessment)	Reduce	Remove	Ineffective, contradicted	Y	Y	Non-empirical	Commentary (literature summary, environmental scan, workshop)

*Note*: *N* no, *NA* not applicable, *NR* not reported, *Y* yes

We identified primary action targets as the focal de-implementation outcome of the FM identified in the included studies. “Remove” was the most common stand-alone action target, occurring in 13 studies (e.g., “deprescribing” of inappropriate medications in older adults [59]). Seven studies sought to “reduce” a practice as a standalone primary action target (e.g., “reducing” unnecessary preoperative testing for low-risk surgical procedures [41]). Six studies had multiple primary action targets, indicating that FM have the power to inform several paths to a de-implementation outcome depending upon the context (e.g., “reduce” dosage or fully “remove” growth hormone therapy in pediatric patients [39]). Eight FM depicted supporting or intermediary steps to reach the primary action target; these were classified as secondary action targets. For example, in a study examining androgen deprivation therapy, restriction (i.e., pre-authorization order templates) was a secondary action to support reduction of this intervention [48].

Fifteen studies provided explicit evidence for the need for de-implementation. Evidence of ineffectiveness was most common (*n* = 12). Five of these 12 studies also cited evidence that previous support for an intervention was contradicted (e.g., Scott and colleagues’ study of de-implementation of polypharmacy interventions for older adults [59]). Because many of the FM included in our review were about de-implementation practices broadly, rather than targeting a specific intervention or policy, we also examined the extent to which authors cited costs or stakeholder input as justification for de-implementation. All but four studies cited specifically cost as a justification for de-implementation. Twenty studies included stakeholder input as part of their rationale for de-implementation; stakeholder input was collected from primary data collection (i.e., surveys, interviews) or cited from other studies (see Tables 1 and 2).

### Model characteristics

In our final analysis, 27 unique FM were found across the 27 included articles. With one exception, the collection of articles from the Harris et al., “SHARE” study [29, 43–45], there was no repetition of models across studies. Process models were the most frequently occurring (*n* = 11), while determinants models were second most common (*n* = 8). Theoretical frameworks (*n* = 5) and evaluation frameworks (*n* = 3) were presented less frequently. Most FM depicted linear relationships between constructs (*n* = 18), suggesting that many authors conceptualized de-implementation activities or processes as a series of steps more often than relationships characterized by feedback or circular processes. Five FM did not depict a relationship between constructs, which may limit their utility in informing de-implementation intervention design or measurement.

Across the 27 studies, 13 unique terms were used to describe the phenomenon of interest. “Discontinuation” and “De-implementation” (and variants of these terms) were used most frequently, appearing across 15 and 13 studies, respectively. Terms that appeared less frequently, such as “deregulate” and “retrenchment” were found exclusively in the public policy literature and did not cross over into other disciplines, whereas other terms from the policy literature, such as “termination” and “disinvestment” were found across disciplines, including within healthcare and public health.

Twenty-one FM encompassed multiple levels of the SEF, indicating de-implementation was conceptualized as a complex and multi-faceted process, while only six operated at a single level. FM most frequently operated at the organization level (*n* = 18). Sixteen included the system level. The intrapersonal and interpersonal levels were represented in 15 and 14 models, respectively. Two of the models that operated at a single level did so at the organization level, while two operated solely at the system level (Table 3).

**Table 3:** Framework and model characteristics

Author	Model name	De-implementation terms used	Relationship between constructs	Socio-ecological framework (SEF)
**Determinants models**				
Amankwah-Amoah, 2017 [51]	Framework of technology obsolescence	Abandon; obsolescence; Terminate	Linear	Organization; system
Cuttler et al., 2005 [39]	Framework for physician decisions to discontinue ongoing medications	Discontinuation; termination	Linear	Intrapersonal; interpersonal
Gupta et al., 2019 [40]	Cost (culture, oversight, systems change, training) framework	Abandon; de-adoption; disinvestment	No relationship indicated	Intrapersonal; Interpersonal; organization; system
Harris et al., 2017b & Harris et al., 2018 [43, 45]	Taxonomy for evaluation and explication of disinvestment project	De-adopt; de-implement; defund; discontinue; disinvestment; obsolescence	No relationship indicated	Intrapersonal; interpersonal; organization; system
Kirkpatrick et al., 1999 [26]	Process model for termination of public goods	Dismantle; retrenchment; termination	Linear	System; policy
Norton et al., 2019 [58]	Continuum of factors influencing de-implementation process	De-implementation; discontinue	No relationship indicated	Intrapersonal; interpersonal; organization; community; system
Padek et al., 2018 [47]	Conceptual framework for mis-implementation	De-adoption; deimplementation; discontinuation; termination	Nested	Intrapersonal; interpersonal; organization; community; system; policy
Skolarus et al., 2018 [48]	Conceptual model for deimplementation of low value prostate cancer care	De-implementation; discontinue	Linear	Intrapersonal; interpersonal; organization; system
**Evaluation frameworks**				
Harris et al., 2017b & Harris et al., 2018 [44, 45]	Framework for evaluation and explication of disinvestment projects	De-adopt; de-implement; defund; discontinue; disinvestment; obsolescence	Linear	Intrapersonal; organization; system
Goodwin, 2013 [40]	Pbma (program budgeting and marginal analysis) evaluation framework	Discontinue; disinvestment	No relationship indicated	Organization
Prasad, 2014 [23]	Potential considerations in prioritizing the testing of unproven medical practice	Abandonment; deimplementation	No relationship indicated	System
**Process models**				
Bain et al., 2008 [52]	Medication use process framework	Discontinuation	Linear; cyclical/feedback	Intrapersonal; interpersonal; system
Davidson et al., 2017 [54]	Virtuous cycle of deimplementation	Abandon; deimplementation	Cyclical/feedback	Intrapersonal; organization
Grimshaw et al., 2020 [41]	Choosing wisely deimplementation framework	De-implementation	Linear	Intrapersonal; interpersonal; organization, system
Helfrich et al., 2018 [37]	Model for de-implementation strategies	De-implementation; discontinue; obsolete	Linear	Intrapersonal
Herald et al., 2019 [55]	Obsolescence management framework	Obsolescence; reassess	Linear	System
Hyun-Ju et al., 2016 [56]	Health technology reassessment process in Korea	Discontinue; disinvestment; obsolescence; reassessment	Linear	Organization; system
McKay et al., 2017 [46]	Implementation framework with EBI de-adoption as a distinct stage	Abandonment; de-adoption; de-implementation; discontinuation; disinvestment	Linear	Interpersonal; organization
Niven et al., 2015 [57]	Synthesis model for the process of de-adoption	De-adoption; discontinue	Cyclical, feedback	Interpersonal; organization
Scott et al., 2013 [59]	Tool for identifying and discontinuing potentially inappropriate drugs	Discontinuation	Linear	Interpersonal
Soril et al., 2020 [60]	Health technology reassessment model	De-adoption; decreased use; disinvestment; obsolescence; reassess	Linear	Interpersonal; organization; system
Voorn et al., 2018 [50]	Grol 2005 implementation model	Abandonment; de-adoption; decrease use; deimplementation; disinvestment	Linear, feedback	Intrapersonal; interpersonal
**Theoretical frameworks**				
Bauer, 2014 [53]	Analytical framework for the explanation of policy dismantling	Abandonment; deregulation; dismantle; retrenchment; termination	Linear	Intrapersonal; system; policy
Harris et al., 2017a & Harris et al., 2018 [29, 45]	Conceptual framework of potential settings and methods to integrate disinvestment into health service systems and processes	De-adopt; de-implement; defund; discontinue; disinvestment; obsolescence	Linear	Organization; system
Harris et al., 2017c & Harris et al., 2018 [44, 45]	Framework for an organization-wide approach to disinvestment in the local healthcare setting	De-adopt; de-implement; defund; discontinue; disinvestment; obsolescence	Nested; linear	Interpersonal; intrapersonal; organization
Tangpong et al., 2015 [49]	Path-dependent pattern of retrenchment and corporate turnaround	Retrenchment	Linear, feedback	Organization

## Discussion

The study of de-implementation is a relatively new area of inquiry within the established and growing field of implementation science [61]. Although a great deal of work in implementation science has been done to gather and classify FM, relatively little is known about the kind of FM that can be applied to study of de-implementation across various settings and topics. This review identified 27 unique FM with visual representations available from a wide range of disciplines, including public health, healthcare, and public policy.

Every study included in this review reported using a unique FM to inform their conceptualization or empirical investigation of de-implementation, suggesting there is considerable variability in topics subject to de-implementation efforts, and potentially in approaches to studying de-implementation. Although we did not see repetition of FM in the records included in this review (with the exception of FM across four articles published from a single study) there were several commonalities across many FM. In particular, we saw that most FM (*n* = 21) operated at two or more levels of the SEF reflecting that de-implementation is often a complex, multilevel process. Four of the six FM that operated at only one level did so at the organization or system level. This could potentially obscure the intrapersonal and interpersonal dynamics that feed into higher-level processes. In addition, we found two-thirds of the FM depicted linear relationships between constructs, while more complex relationships (e.g., feedback loops) were depicted less frequently. This simplified illustration of relationships between constructs or processes involved in de-implementation may dampen the extent to which real-world complexity is conceptualized and considered. Indeed, a review of low-value health services found that multicomponent interventions involving patients and providers were most effective in reducing low-value care [62], suggesting that the study of de-implementation warrants multilevel FM that depict these various components of a de-implementation effort.

As previously stated, apart from a collection of articles stemming from a single study [29, 43–45], we did not see FM repeat across studies included in our review, which may be due in part to the variety of settings and interventions included. The lack of congruence in conceptualization of de-implementation determinants and processes may make it difficult to compare findings across studies. The use of existing FM would allow for comparisons across studies, testing and confirmation of proposed relationships between variables related to de-implementation efforts, and offers opportunities for refinement and improved conceptual clarity within the study of de-implementation processes [63, 64]. As research focusing on de-implementation advances, we suggest that researchers begin with models reviewed here, with necessary adaptations, to help promote congruence and convergence within the field.

Only three of the FM included in our review were also included in a recently published review of de-implementation for low-value care [32]. Ultimately, we included 27 unique articles, compared to 10 included by Nilsen and colleagues. Despite differences in our review approach highlighted earlier, we note several similar findings. In both reviews, authors often did not specify the setting of interest; just over half of studies specified to what kind of clinical (or non-clinical) setting their FM applied. On the surface, broad descriptions of the settings may seem to suggest that the included FM can be applied to a wide array of contexts. However, we are limited in determining the extent to which FM developed in one setting or for a particular topic can be successfully applied to another substantive area (for example, would FM developed in the context of reducing medication overprescribing be suitable for discontinuation of a psychosocial intervention, or would FM developed for use in hospitals also be applicable in community settings). Across both reviews, samples were roughly evenly split between conceptual and empirical studies. This could suggest that, despite dating back as much as two decades, many proposed FM have yet to be empirically applied. This illustrates the need for additional testing and refinement of existing de-implementation FM.

Reducing low-value care can help improve health equity [18]. Cost savings from improved health care delivery can be used for addressing needs related to social determinants of health, expanding healthcare benefits to uninsured patients, and bolstering safety net care [21]. There is opportunity to more intentionally build an equity lens into de-implementation FM. Indeed, the revision and expansion of well-established implementation FM (e.g., RE-AIM, iPARIHS) to include health equity is already underway [65, 66]. Similar approaches can be emulated with de-implementation FM. There is also opportunity to harness de-implementation to improve health equity; however, caution is needed in ensuring that de-implementation efforts do not unintentionally perpetuate or exacerbate existing inequities [22]. For example, in the context of de-implementing outdated cancer screening guidelines, Shelton and colleagues note several important findings related to de-implementation and impacted stakeholders, including mistrust of healthcare systems among patients and misalignment of changing guidelines with the preferences and needs of African American women [67]. Finally, all studies for which the location of the work was reported (20 of 27 studies) were conducted in OECD counties. It is unclear the extent to which models developed in OECD contexts are applicable to low- and middle- income countries, or what lessons from de-implementation in these countries can be applied to resource-limited settings in high-income countries. More work is needed in conceptualizing and examining de-implementation in resource-limited locations.

Implementation science is an interdisciplinary field [68, 69], yet its use of FM has not taken advantage of the theoretical depth that exists in its associated disciplines. Implementation researchers have argued both for better integration of knowledge from other disciplines, including public administration and economics [70, 71], and improving implementation science’s use and development of theory [68]. Many analyses highlight the ability of public policy to better address the complexity of implementation, clarify the role of the outer context, conceptualize a broader range of policy outcomes, contribute to development of strategies at the systems level, and measure policy implementation determinants and outcomes [72–74]. Similar integration could serve as a way to advance the study of de-implementation by taking advantage of the comparatively large policy termination literature. Classic theories of policy feedback and incrementalism [75, 76] have proven particularly useful to de-implementation research in that they detail how the political landscapes change after a policy is adopted and sustained for some time, leading to distinct risks and barriers of termination of these policies than at adoption.

Although interest in de-implementation is growing, opportunities to advance this area of inquiry remain few [77]. Through 2016, only 20 federally-funded grants from the National Institutes of Health and the Agency for Healthcare Research and Quality focused explicitly on de-implementation [78]. Moullin and colleagues suggest several purposes and benefits of using FM to inform research and practice, including: defining the issue of interest and developing research or evaluation questions, selecting appropriate research or evaluation methods, discerning relevant determinants to de-implementation, selecting and tailoring strategies to support de-implementation, and to specify key outcomes to target [63]. Implementation science is a field that draws upon diverse disciplines, and there are numerous examples of FM that have been developed by scholars from a variety of fields (e.g., Theoretical Domains Framework developed by implementation scientists and behavioral researchers [79]). However, future work is still needed to determine the extent to which FM from other fields (e.g., cognitive psychology, organizational behavior) can be applied to de-implementation of healthcare interventions and public health policies and programs, as well as the transferability of FM from one content area to another (e.g., diabetes to cancer or prevention to treatment). Furthermore, additional investigation is needed to determine the extent to which FM for implementation are applicable to de-implementation. There may be opportunities to learn from previous implementation FM development work and to adapt implementation models for de-implementation. The field would benefit from a publicly available repository of de-implementation FM and associated literature, such as the resource available here: https://dissemination-implementation.org/.

We offer potential approaches for researchers and practitioners to use the information provided in this review. Readers seeking to use an existing FM to guide de-implementation work may wish to define their action target based on the categories used in this review (reduce, replace, restrict, remove) and select models that were applied to the same type of action. Readers may also wish to identify the level(s) of the SEF at which their intervention or approach to de-implementation operates and select from frameworks that operate at these level(s). Finally, as this review identifies over a dozen FM proposed in conceptual papers but (to our knowledge) have not been applied to empirical studies of de-implementation, readers may wish to test and refine FM identified in this review. The purpose of this review is to offer a broad understanding of existing FM to guide the study and practice of de-implementation; scholars can build upon this work to further specify and analyze de-implementation FM.

### Limitations

While effective in some ways, the approach used in this review carries some limitations. Because visual representation of a FM (e.g., figure, table) was a requirement for inclusion, empirical studies that cited use of a FM but did not depict it, or for which we could not find a visual depiction in any of the literature referenced, were excluded. This approach was taken to minimize ambiguity of relationships between constructs and to include potentially helpful resources that can be used in future de-implementation studies. It is worth noting that many of the FM that did not have a visual depiction were prominent implementation frameworks that were not developed explicitly for the study or practice of de-implementation, thus would have been excluded from this review even with a visual representation. Further investigation is needed to determine the characteristics of other FM in existence that have been developed for the purpose of de-implementation yet do not yet have a visual representation available in the literature. These may offer additional opportunities to build upon existing work to develop, test, and refine de-implementation FM.

Although we conducted an extensive search incorporating seven databases and 11 peer-reviewed journals, our search did not yield a high number of records related to public policy de-implementation. It is possible that our search strategy was not inclusive of databases that include more policy-focused articles, or that relevant policy frameworks appear in books and other texts rather than peer-reviewed journals. Additionally, our single rater approach at the title and abstract screening phase may have resulted in relevant records being excluded. However, we limited our exclusion criteria at this phase and erred on the side of including records for full text screening if additional information was needed to determine article relevance. Finally, it was beyond the scope of this current review to conduct systematic coding and content analysis of the individual constructs in each of the FM. A future systematic review would be valuable in serving this purpose, and would allow for additional analyses, such as synthesis of the common issues identified across FM. It will also be useful to add de-implementation FM to webtools for finding, adapting, and using FM in implementation science [80].

## Conclusions

This current review highlights the growing interest in the study of de-implementation and provides a broad overview of FM that can be applied to this area of inquiry. As we continue to build the de-implementation knowledge base, the field of implementation science, as well as implementation practitioners, would benefit from the greater specification of how FM are applied to de-implementation challenges. The use of identified de-implementation FM would enable researchers to make comparisons across studies and contribute to a knowledge base regarding key constructs related to de-implementation and the relationships between these.

## Supplementary Information

**Additional file 1.** Study identification and screening.

**Additional file 2.** De-Implementation Review Data Extraction.

**Additional file 3:** Quality assessment of empirical studies (*N*=13).

## Data Availability

PDFs of articles included for data extraction and raw extraction data tables can be provided by the authors upon reasonable request.

## Abbreviations

FM: Frameworks and models
SEF: Socioecological framework
NR: Not reported
