# Supplementary material for: A scoping review of de-implementation frameworks and models
Source: Implement Sci. 2021 Nov 24;16:100. doi: 10.1186/s13012-021-01173-5 (PMC8611904; doi:10.1186/s13012-021-01173-5)
Supplement: Supplementary file 1 — Additional file 1. Study identification and screening. [file 13012_2021_1173_MOESM1_ESM.docx]

**Search Strategy:**

Limiters: PY = 1990-current; types (all); human studies only/no animal; English language

Database –Medline/Ovid + Embase + CINAHL + EBSCO(Econlit, Global Health, APA PsycInfo, Political Collection Index, Political Science Database) + ProQuest + Cochrane Library + Scopus)

Search strategies used

| Action | Term |
| --- | --- |
| 1 | (('deimplement*' or 'de-implement*') adj2 ('model*' or 'framework' or 'barrier' or 'facilita*' or 'approach' or 'polic*' or 'intervention*')).ab,kw,ti |
| 2 | (('deadopt*' or 'de-adopt*') adj2 ('model*' or 'framework' or 'barrier' or 'facilita*' or 'approach' or 'polic*' or 'intervention*')).ab,kw,ti |
| 3 | (('disinvest*' or 'abandonment') adj2 ('model*' or 'framework' or 'barrier' or 'facilita*' or 'approach' or 'polic*' or 'intervention*')).ab,kw,ti |
| 4 | (('obsoles*' or 'discontinuation') adj2 ('model*' or 'framework' or 'barrier' or 'facilita*' or 'approach' or 'polic*' or 'intervention*')).ab,kw,ti |
| 5 | (('reassess*' or 'decreased use') adj2 ('model*' or 'framework' or 'barrier' or 'facilita*' or 'approach' or 'polic*' or 'intervention*')).ab,kw,ti |
| 6 | (('terminat*' or 'dismantl*') adj2 ('model*' or 'framework' or 'barrier' or 'facilita*' or 'approach' or 'polic*' or 'intervention*')).ab,kw,ti |
| 7 | (('retrench*' or 'deregulat*') adj2 ('model*' or 'framework' or 'barrier' or 'facilita*' or 'approach' or 'polic*' or 'intervention*')).ab,kw,ti |
| 8 | (('de-fund' or 'defund*') adj2 ('model*' or 'framework' or 'barrier' or 'facilita*' or 'approach' or 'polic*' or 'intervention*')).ab,kw,ti |
| 9 | Or/1-8 |
| 10 | exp Practice Patterns, Physicians'/ |
| 11 | exp program evaluation/ |
| 12 | ('practice patterns' or 'use patterns' or 'applications' or 'low-value practice*' or 'clinical practice*' or 'low-value care' or 'programs' or 'procedures' or 'strateg*').ab,kw,ti |
| 13 | OR/10-12 |
| 14 | 9 AND 13 |
| 15 | 14 (english language and yr="1990 -Current") |

2. Protocol search

Targeted search of journals publishing study protocols:

| BMC Health Services Research | Implementation Science Communications |
| --- | --- |
| BMC Medicine | Journal of European Public Policy |
| BMC Public Health | Review of Policy Research |
| Contemporary Clinical Trials | Social Science and Medicine |
| Health Policy | Trials |
| Implementation Science |  |

**Inclusion/Exclusion Criteria**

Inclusion criteria: Models/frameworks developed for de-implementation

Exclusion criteria: Models/frameworks not developed for de-implementation

|  | Include | Exclude |
| --- | --- | --- |
| Year Published | 1990 or later | Before 1990 |
| Language | Original article published in English or full-text English translation available | Non-English |
| Model or Framework | - Study of de-implementation directly informed by model or framework - Visual representation of model or framework is available in the form of an image, figure, or table | - Model or framework referenced in passing, but does not directly inform study design, or role of model unclear - Model or framework present, but is not related to deimplementation - Model informed study design, but no visual representation of the model is available (in a figure or table) |
| Search terms | - Terms refer to de-implementation, de-regulation of an intervention, guideline, policy, or practice | - Search terms used in a different context (e.g. gene” “de-regulation”, RNA transcription “termination”, statistical “model” |

| **E Codes Exclusion codes** – at full text screening  **Hierarchy top down per where in article likely to find the information** |
| --- |
| **E1.** Irretrievable |
| **E2**. Not English |
| **E3.** Publication date pre-1990 |
| **E4.** Model or framework not depicted  **E4a.** Study does not use a model or framework  **E4b.** Study references a model or framework, but does not provide a visual representation  **E4c.** Model or framework not relevant to de-implementation |
| **E5.** Search term used in a different context (e.g. gene de-regulation, transcription termination) |

| **X Codes Supplemental codes –**  **used to flag articles of interest** |
| --- |
| **X1.** Check cited references |
| **X2.** Check for papers citing this one |
| **X3**. Named model or framework; check for other papers using |
| **X4**: Introduction or discussion |
