# Supplementary material for: A scoping review of de-implementation frameworks and models
Source: Implement Sci. 2021 Nov 24;16:100. doi: 10.1186/s13012-021-01173-5 (PMC8611904; doi:10.1186/s13012-021-01173-5)
Supplement: Supplementary file 2 — Additional file 2. De-Implementation Review Data Extraction. [file 13012_2021_1173_MOESM2_ESM.docx]

**Appendix B: De-Implementation Review Data Extraction**

| Variable | Description | Example |
| --- | --- | --- |
| **Section 1: Study description** | | |
| **A. Author** | Author last name will be populated in the extraction sheet based on articles identified during full text review | *Smith* |
| **B. Year** | Year of article publication will be populated in the extraction sheet based on articles identified during full text review | *2018* |
| **C. Setting** | Setting in which de-implementation study was conducted. If the conceptualization is broad or studies conducted in multiple settings, list each setting or indicate “broad” | *Hospitals; state-level government* |
| **D. Country** | Indicate the country in which the study was conducted. If multiple countries in the same continent, aggregate to the continent level. If multiple continents represented, type each continent  If a conceptual/commentary paper, list countries main examples are from | *Canada;*  *Europe* |
| **E. Topic/content area** | Topic or content area of de-implementation intervention. If the conceptualization is broad, indicate the general field (e.g. healthcare, agriculture) | *Gastroenterology; school nutrition* |
| **F. De-implementation intervention** | Describe the specific policy or practice to be de-implemented. Describe the process(es) or strategies used to deimplement the policy, practice, etc (include approach for selecting the de-implementation strategy). Provide specific details about the de-implementation strategy (e.g. goal setting, provider feedback) when available, and general de-implementation process (e.g. behavior change) | *Provider education and behavior change strategy to reduce inappropriate medication prescribing; changing cafeteria menu guidelines in terminating a school nutrition policy* |
| **G. Primary Action** | This is the main de-implementation outcome of interest (e.g. endpoint in a linear model, or outcome most emphasized in a cyclical model). Categorize the specific de-implementation action taking place as: **remove** (stop, discontinue), **replace** (with something better), **reduce** (frequency or intensity/dosage), **restrict** (universal screening vs. high risk) | *Remove* |
| **H. Secondary Action** | These are any actions that support the ultimate de-implementation outcome, but are not the main focus or end-point of a model. Categorize the specific de-implementation action taking place as: **remove** (stop, discontinue), **replace** (with something better), **reduce** (frequency or intensity/dosage), **restrict** (universal screening vs. high risk) | *Replace* |
| **I. Evidence for De-Implementation** | Categorize the evidence related to the subject of de-implementation as (can indicate >1): **ineffective** (practice is ineffective in eliciting desired outcome, evidence that practice is harmful), **contradicted** (new evidence suggests practice is ineffective or harmful), **mixed** (no clear consensus on effectiveness), **untested** (intervention not sufficiently tested for effectiveness), **other** (describe the evidence that does not fit one of the previous options), **NR** (empirical evidence not cited) | *Contradicted and ineffective* |
| **J. Cost** | Use dropdown to select y/n for authors cite cost as a supporting reason for de-implementation (e.g. current practice is more costly than other equally effective practice) | *yes* |
| **K. Stakeholder** | Use dropdown to select y/n for stakeholder input was included in the decision to de-implement (e.g. a focus group of providers, comments from patients) | *no* |
| **L. Method** | Indicate quantitative, qualitative, mixed methods, or review; select non-empirical if primary data collection not used | *Qualitative* |
| **M. Study Design** | Type a brief description of the study design, including randomization, time points (i.e. longitudinal vs. cross-sectional), etc. List the paper type if not an empirical study and describe the approach used | *Commentary (literature search, expert consensus);*  *Non-randomized, longitudinal* |
| **N. Measures Used** | Describe measure(s) used (e.g. survey, observation checklist, interviews, focus groups, chart/EHR abstraction, secondary review of policy database). If a named measure or scale was used, indicate the name | *Focus group; EBPAS (survey); ORCA (survey); Food-Epi (policy checklist)* |
| **O. Primary Outcomes** | Describe the primary implementation outcome of interest and/or primary clinical outcome of interest | *Acceptability of reducing upper GI endoscopy; 20% reduction in antibiotic prescribing* |
| **Section 2: Categorization of models and frameworks** | | |
| **Model Name** | Title of model or framework if named; otherwise, use author last name, year, and brief description  Will be populated in the extraction sheet based on articles identified during full text review | *Bauer 2014 Policy termination model* |
| **De-Implementation search terms used** | Indicate which relevant terms the article uses to describe the study of the phenomenon of interest (de-implement*, de-adopt*, disinvest, abandon*, obsoles*, discontinue*, reassess*, decreased use, terminat*, dismantle*, retrench*, deregulat*, de-fund) | *De-adopt;*  *termination* |
| **Other de-implementation terms used** | List any terms beyond those in the search strategy that describe de-implementation (e.g. extinct, extinguish, remove) | *Remove* |
| **Model Type** | **Determinants model** (provides contextual factors that could change or influence implementation; example: CFIR); **process model** (provides a step by step “how to” approach; example: Plan-Do-Study-Act); **evaluation framework** (used to evaluate or assess interventions; example: RE-AIM); **theoretical framework** (depicts relationships between constructs proposed in formal theories that are structured, technical, and provide formal definitions or operationalization of constructs; example: transtheoretical model) | *Process model* |
| **Relationship between constructs** | No relationship indicated; linear; cyclical/feedback; nested | *Linear and feedback* |
| **Socio-ecological**  **Framework (SEF)** | Level of the SEF at which the model operates: **Intrapersonal** (internal behavior change, personal characteristic); **Interpersonal** (interactions between individuals or groups of people); **Organization** (internal operations of characteristics of single organizations e.g. hospitals, factory); **Community** (e.g. neighborhood, network of individuals and organizations in a city); **System** (e.g. healthcare system); **Policy** (e.g. national child nutrition policy) | *Interpersonal (providers & patients), Organization* |
