# Supplementary material for: A scoping review of de-implementation frameworks and models
Source: Implement Sci. 2021 Nov 24;16:100. doi: 10.1186/s13012-021-01173-5 (PMC8611904; doi:10.1186/s13012-021-01173-5)
Supplement: Supplementary file 3 — Additional file 3: Quality assessment of empirical studies (N=13). [file 13012_2021_1173_MOESM3_ESM.docx]

**Supplemental File 4: Quality assessment** **of empirical studies (N=13)**

|  |  | Cuttler, 2005 [43] | Goodwin, 2013 [68] | Grimshaw, 2020 [42] | Gupta, 2019 [69] | Harris, 2017a [29] | Harris, 2017b [46] | Harris, 2017c [47] | Harris, 2018 [48] | McKay, 2017 [70] | Padek, 2018 [71] | Skolarus, 2018 [44] | Tangpong, 2015 [39] | Voorn, 2018 [40] |
| --- | --- | --- | --- | --- | --- | --- | --- | --- | --- | --- | --- | --- | --- | --- |
| Screening questions | Clear research questions | Y | Y | Y | Y | Y | Y | Y | Y | Y | Y | Y | Y | Y |
|  | Collected data address the research questions | Y | Y | Y | Y | Y | Y | Y | Y | Y | NA | NA | Y | Y |
| Mixed methods | Adequate rationale for using a mixed methods design | Y | Y | Y | Y | Y | Y | Y | Y | Y | Y | Y |  |  |
|  | Different components of the study effectively integrated | Y | Y | C | Y | Y | Y | Y | Y | Y | Y | Y |  |  |
|  | Outputs of the integration of qualitative and quantitative components adequately interpreted | N | Y | C | Y | Y | Y | Y | Y | Y | C | C |  |  |
|  | Divergences and inconsistencies between quantitative and qualitative results adequately addressed | N | Y | C | N | N | N | N | N | Y | C | C |  |  |
|  | Different components of the study adhere to the quality criteria of each tradition of the methods | Y | Y | C | Y | Y | Y | Y | Y | Y | Y | Y |  |  |
| Quantitative descriptive | Sampling strategy relevant to address the research question |  |  |  |  |  |  |  |  |  |  |  | Y |  |
|  | Sample representative of the target population? |  |  |  |  |  |  |  |  |  |  |  | Y |  |
|  | Measurements appropriate |  |  |  |  |  |  |  |  |  |  |  | Y |  |
|  | Risk of nonresponse bias low |  |  |  |  |  |  |  |  |  |  |  | Y |  |
|  | Statistical analysis appropriate to answer the research question |  |  |  |  |  |  |  |  |  |  |  | Y |  |
| Quantitative randomized controlled trials | Randomization appropriately performed |  |  |  |  |  |  |  |  |  |  |  |  | Y |
|  | Groups comparable at baseline |  |  |  |  |  |  |  |  |  |  |  |  | Y |
|  | Complete outcome data |  |  |  |  |  |  |  |  |  |  |  |  | Y |
|  | Outcome assessors blinded to the intervention provided |  |  |  |  |  |  |  |  |  |  |  |  | N |
|  | Participants adhere to the assigned intervention |  |  |  |  |  |  |  |  |  |  |  |  | Y |
|  | Total (out of 5) | 3 | 5 | 1 | 4 | 4 | 4 | 4 | 4 | 5 | 3 | 3 | 5 | 4 |

*Note: Y = Yes; N = No; C=Can’t tell; NA = Not applicable*; Full screening criteria and screening guidelines available from Hong et al., 2018 [38].
